# Urolithiasis treatment options during COVID-19 pandemic: review of current recommendations and triage systems

**DOI:** 10.1186/s12301-020-00085-y

**Published:** 2020-11-25

**Authors:** Ali Abdel Raheem, Ibrahim Alowidah, Mohamed Soliman, Mefarrih Haresy, Ali Almozeni, Sultan Althagafi, Mohamed Almousa, Mohamed Alturki

**Affiliations:** 1 Department of Urology, Faculty of Medicine, Tanta University, Tanta, Egypt; 2 Department of Urology, King Saud Medical City, Riyadh, Saudi Arabia

**Keywords:** Urolithiasis, COVID-19, Pandemic, Recommendations

## Abstract

**Background:**

COVID-19 pandemic has overwhelmed healthcare systems and limited access to surgical care. Urolithiasis can lead to emergencies and affect renal function during long-term follow-up. Therefore, timely and appropriate treatment is essential.

**Main text:**

This is a non-systematic review of the recently published recommendations regarding urolithiasis treatment options during COVID-19. Fourteen publications were the basis of our review. Regarding anesthesia methods, the optimal methods are still unknown. During COVID-19, most of the endo-urologists changed their routine clinical practice and elective surgical treatment approaches. Despite decreasing number of emergency visits and admissions for stone disease, patients tend to have leukocytosis, higher creatinine levels, increased grade 3 and 4 hydronephrosis, and higher incidence of complications compared to non-COVID-19 time. Several alarming indications if present, intervention should be performed within 24 h to prevent irreversible kidney damage, disease progression, or even death. Some endo-urologists prefer definitive stone treatment over temporarily drainage to reduce the number of emergency room visits and hospital admissions, except if infection is present or staged treatment is planned. Several clinical scenarios of non-emergency and non-urgent urinary stones are present; thus, endo-urologists should appropriately weigh patient’s risk and surgery benefit to decide to the proper intervention time. If risks outweighed benefits to the patient, postpone the surgery. Renal colic should be managed with medical expulsive therapy and proper pain control with close follow-up just in case it becomes an emergency. Indwelling JJ stent removal or exchange is a matter of debate; some endo-urologists recommend removing, while others recommend postponing.

**Conclusion:**

Treatment options for urinary stones have markedly changed during COVID-19 pandemic. The optimal anesthesia methods are still unknown. Emergency intervention is a must if any alarming indications exist. Emergency cases tend to have higher incidence of complications compared to non-COVID-19 time. For non-emergency and non-urgent urolithiasis, endo-urologists should make judicious treatment decision to prioritize urolithiasis treatment, and they should weigh benefits and risks before surgery.

## Background

In March 2020, the World Health Organization (WHO) declared COVID-19 as a global pandemic. At the time of writing, more than 12 million cases and 550,000 deaths have been reported [1]. Unfortunately, COVID-19 has overwhelmed healthcare systems of all countries, depleted hospitals’ resources, reduced the provision of medical services, and limited access to surgical care.

The USA is the most affected country with COVID-19 pandemic in the world, with an estimate of 3,098,084 reported cases and 133,972 total deaths on July 7, 2020 [1]. Subsequently, hospitals workload slashed by more than 50% during this unprecedented emergency scenario, as well as urology workload slashed by 72%. Moreover, the monthly estimated financial losses of USA hospitals reached up to 60 Billion $ [2].

In Europe, Italy was severely affected with COVID-19 pandemic, especially in March and April 2020. In Bergamo City, the marked surge of virus infection and increase in the number of critically ill patients led to a reduction in beds’ capacity by two-thirds, and at certain time, urological procedures including some emergency surgery were stopped completely due to lack of resources and medical staff [3].

Urolithiasis is a unique disease that can lead to emergencies and can adversely affect kidney function during long-term follow-up, especially if infections coexist. Therefore, timely and appropriate management is essential. Recently, Flammia et al. studied the change of urinary stone emergencies in the time of COVID-19. Their findings suggested that urinary stone emergencies are mainly severe, as patients presented during COVID-19 pandemic had higher levels of serum creatinine compared to a non-COVID-19 time, and continuous care should be maintained for those patients [4].

We aimed in the current review to give a critical insight into the recently published recommendations, clinical pathways, and triage systems of the different treatment options for urinary stones in the time of COVID-19, to help urologists in their treatment decision during this unprecedented situation.

## Main text

### Materials and methods

In this non-systematic review, we searched articles in PubMed from January 1, 2020, through June 1, 2020. We used the search terms “COVID-19,” “Coronavirus,” “SARSCoV-2,” “Pandemic,” “Urinary stones,” “Urolithiasis,” “Treatment,” “Triage,” “Guidelines,” and “recommendations.” We included original articles, review articles, research letters, letter to editors, commentaries, and editorials. Non-English language articles were not included, as well as COVID-19 researches that were out of scope to our research.

Finally, fourteen publications were the basis of our review article. Most of evidence in the present review is based on experience of the authors in the management of COVID-19 in their institutions.

## General considerations for urolithiasis treatment options during COVID-19

### Impact of COVID-19 on urolithiasis practice

Urolithiasis practice pattern has markedly changed during COVID-19. According to the EULIS Collaborative Research Group, a large survey that included 60 physicians whose main area of expertise is urinary stones was conducted to evaluate urolithiasis practice patterns following the COVID-19 pandemic. The survey showed that 49% of experts experienced > 90% change in their routine clinical practice. Among them, 72.3% used telemedicine during the crisis. 89.4% of the responders tended to change the treatment strategy of emergency COVID-19 patients by planning temporary collection system drainage followed by an elective intervention afterward. Nevertheless, 10.6% of them continued to perform definitive stone surgical treatment. It is worth noting that 55.3% and 39.8% of the experts changed their elective surgical treatment approaches by a rate of 90–100% and 75–89%, respectively. On the other hand, 6.4% of them continued as before the pandemic [5].

Antonucci and colleagues studied the impact of COVID-19 outbreak on urolithiasis emergency department (ED) admissions, hospitalizations, and clinical management in three high-volume Italian centers. Among 304 patients included in the analysis, there was a significant reduction (48.4%) in the global number of patients admitted to ED for treatment of urolithiasis between March and April 2020 compared to the same period of the last year. Moreover, patients admitted to ED during COVID-19 had more complications (20.4% vs. 10.9%, *p* = 0.025), more frequently need hospitalization (38.8% vs. 20.9%, *p* = 0.001), and regarding clinical stone management a statistically significant increase in early stone removal procedures over urinary drainage only was reported (*p* = 0.015) [6]. Likewise, in Dallas, USA, Steinberg and colleagues observed a 38% and 44% reduction in the number of ED visits for stone disease at both their private academic and county hospitals, respectively [7].

In several hospitals, it took about 21 days to adopt changes related to COVID-19 and intervention for urinary stones. There was a significant increase in the rate of conservative approaches such as nephrostomy tube (NPT) insertion, double JJ stent placement or extraction from 38.2 to 81%, while definitive treatment options such as ureteroscopy (URS), retrograde intrarenal surgery (RIRS), and percutaneous nephrolithotomy (PCNL) dropped from 60.8 to 19% (*p* < 0.001) [8].

In another study that compared the diagnostic and therapeutic procedures for management of urinary stone emergencies during COVID-19 pandemic (i.e., March–April 2020) with the management performed in the same hospital in a non-COVID-19 period (i.e., March–April 2019), the number of urinary stone emergencies, complication rates, urinary stone diameter, grade of hydronephrosis, and the use of NPT or ureteral stent for the first aid did not significantly change during COVID-19 pandemic [4]. However, patients had higher serum creatinine levels and stone position significantly changed with increase rate of middle and lower third ureteric stones during COVID-19 time due to delay of patient presentation to the hospital, related to the pandemic [4]. Similarly, Gul and colleagues found that serum creatinine levels and the white blood cell counts at hospital admission were significantly higher in the COVID period and the rate of grade 3 and 4 hydronephrosis was higher. These findings reflect the increased rate of complicated ureteral stone disease during the COVID-19 restrictions period [9].

### Recommendations, triage systems, and clinical pathways

Recently, several researchers have published recommendations to prioritize the treatment of urinary stones during the COVID-19 pandemic [10–20]. Ribal and colleagues divided the priority of urological diseases into: low priority (if treatment delayed by 6 months, it is unlikely to cause clinical harm); intermediate priority (if treatment delayed by 3–4 months, it may cause clinical harm, but it is unlikely); high priority (if treatment delayed more than 6 weeks, it is likely to cause clinical harm); and emergency (a life or organ-threatening situation) [10]. Others have developed triage tier classification systems [11, 13] and clinical pathways [12] to facilitate decisions on surgical care of patients with urinary stones. Tier systems ranged from “tier 0 to tier 4” based on the urgency to intervene. Tier 0 was classified as top emergency cases that require intervention within 24 h, whereas tier 4 can be postponed >12 weeks.

### Factors affecting urolithiasis treatment decision

With regard to strategies for the prevention and treatment of urinary stones during this COVID-19 pandemic, patients can be divided into two groups. First group includes those who do not need urological intervention including non-struvite, non-cystine renal stones < 7 mm, with no anatomic abnormalities. In this group of patients, general dietary recommendations and lifestyle modifications are helpful, and it is preferred to perform follow-up ultrasonography after cessation of the COVID-19 pandemic. The second group comprises those patients in whom urological intervention either emergent or nonemergent is indicated [19]. There are multiple parameters used to assess the urgency of surgical intervention for treatment of urolithiasis including stone size and site, severity of symptoms, control of symptoms, presence of hydronephrosis or infection, degree of obstruction, presence of indwelling JJ stent or nephrostomy tube (NPT), and if the patient has a solitary functioning kidney and/or renal function impairment [11–19].

Of note, the treatment decision of urinary stones treatment depends not only on the patient and calculus-related factors, but also on other disciplines and healthcare resources, including the number of surgical staff and anesthesiologists, availability of hospital beds, operating rooms, and mechanical ventilators, as well as the burden of COVID-19 in the country.

### Preoperative evaluation and anesthesia applied for urinary stones

Medical staff are at risk of contracting COVID-19 infection from positive diagnosed COVID-19 patients, asymptomatic or patients in the incubation period. Anesthesiologists have more risk of contracting infection during intervention from exposure to the patient’s airway [21]. Regional anesthesia may provide patients with a successful anesthesia method and help protect the anesthesia team [22]. Nevertheless, recent report showed that COVID-19 virus may also spread the virus during regional anesthesia as it can affect the central nervous system [23]. Thus, it is necessary to determine the principle of the best preoperative evaluation during a pandemic to protect healthcare workers.

Recently, Gökce et al. studied the preoperative evaluation and methods of anesthesia applied for stone disease treatment during COVID-19 pandemic. They included 473 patients from 11 centers in 5 countries, and they found CT chest scan and PCR from the nasopharyngeal swab increased by 59.6% and 56.7%, respectively. In addition, there was significant alteration in anesthesia methods by 9.5%. General anesthesia, spinal/epidural anesthesia, and topical/local anesthesia were applied in 71.2%, 16.1%, and 11% of patients, respectively [8].

## Treatment options and urgency to intervene

### Emergency urolithiasis (intervention within 24 h)

For treatment of obstructed renal or ureteric stones, all studies have assigned alarming indications and warning signs for intervention within <24 h, including infection, impaired renal function, solitary kidney, bilateral ureteric obstruction, and intractable symptoms, in order to prevent irreversible kidney damage and disease progression, or even death [11–19]. The ideal intervention time in case of treatment of longtime ureteric and renal obstruction is not determined yet, because several unpredictable variables affect the dynamics of renal function loss [24]. If obstruction is not associated with urinary tract infection, the time frame of intervention between 6 and 12 weeks seems suitable; however, if infection is superadded, immediate intervention is required to avoid any possible renal function loss [25]. Of note, delayed intervention for obstructed infected kidney might increase the risk of ICU admissions and mortality rates by 15% and 8%, respectively [26].

### Temporarily drainage versus definitive treatment

If obstruction is associated with infection and fever, we should drain the collecting system temporarily using either indwelling JJ stent or NPT, followed by definitive treatment when possible [12, 13, 15, 16, 18]. During COVID-19 pandemic, definitive stone treatment is still a matter of debate. Some endo-urologists prefer active stone treatment over temporary drainage to reduce the number of emergency room visits, except if infection or staged treatment is expected [6, 13, 15], while others prefer to defer all procedures to treat urinary stones until the end of the COVID-19 pandemic, with temporary drainage only if indicated [16, 18].

### Non-emergency urolithiasis (intervention from 2 to 12 weeks)

**Fig. 1:**
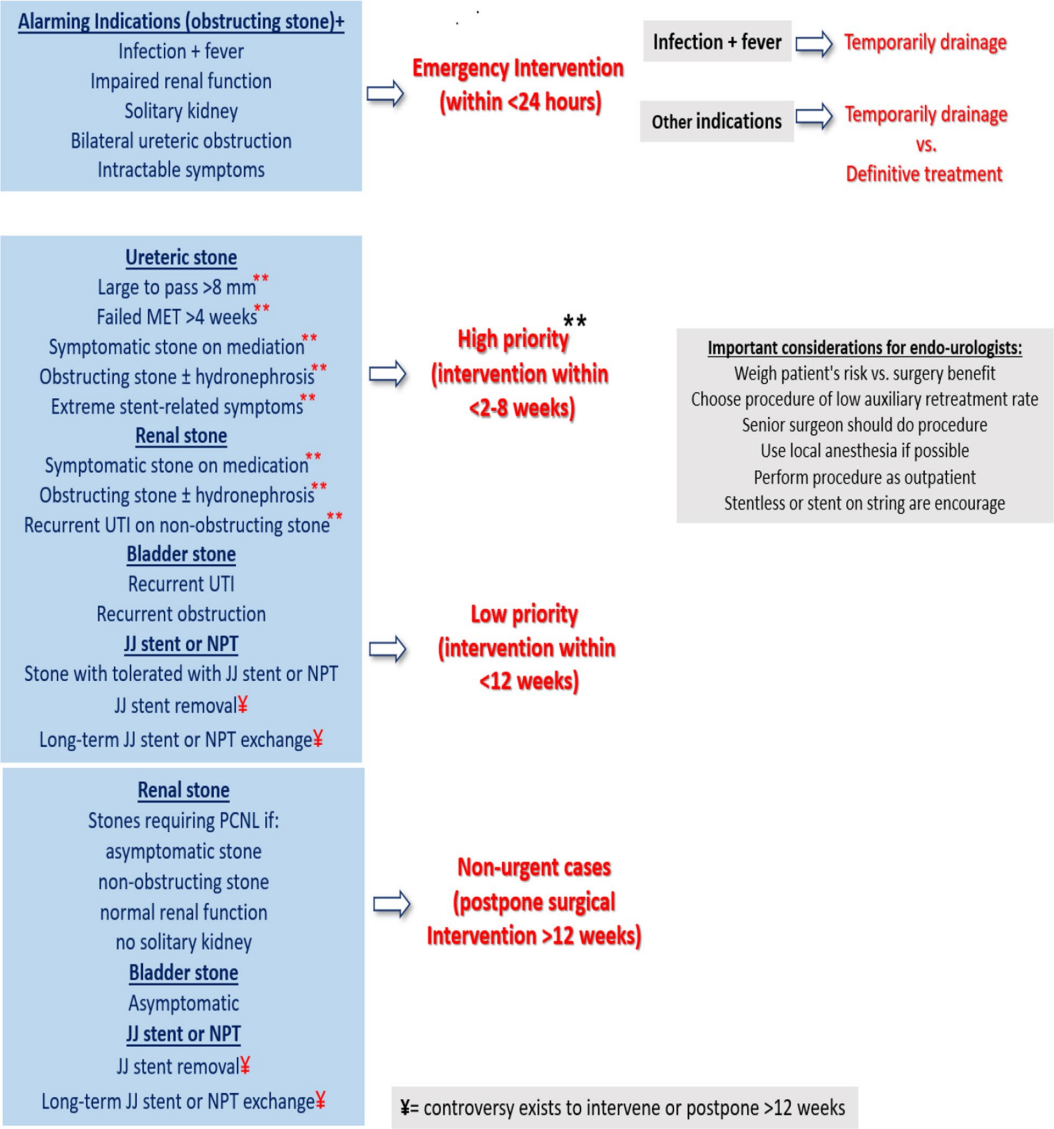
Algorithm that summarized current recommendations and triage systems for treatment of urolithiasis during COVID-19

As shown in Table 1 and Fig. 1, there is a wide range of clinical scenarios to determine the appropriate intervention time, because if delay in intervention happens, clinical harm is likely to occur. Thus, endo-urologists should appropriately weigh the patient’s risk and the benefit of the surgery to decide to intervene. If the risks outweighed the benefits to the patient, postpone the surgery. Moreover, it is advisable that endo-urologists should choose the treatment option that achieves the higher stone-free status and has a lower auxiliary treatment rate. For example, for treatment of stone upper third left ureter URS is recommended than SWL [13].

**Table 1:** Current recommendations and triage systems for treatment of urolithiasis during COVID-19

Urgency classification	Definition [7, 8, 10]	Time to intervene [10, 11, 13]	Indications	Important considerations
Emergency or Tier 0 [10, 11, 13]	Organ-threatening or life-threatening	< 24 h	Obstructed infected kidney [11–18, 20] Obstructing stone in solitary kidney [12–15, 20] Obstructing stone associated with acute renal impairment [12–15, 20] Bilateral ureteric obstruction [12–15, 20] Severe unmanageable symptoms [12, 13, 15, 20]	Offer temporarily drainage if infection and fever present Consider definitive treatment, except if staged treatment is expected
High priority or Tier 1–3 [10, 11, 13]	It is likely to cause clinical harm	< 2–8 weeks	Obstructing ureteric stone if failed MET (> 4 weeks), large to pass (> 8 mm) [13] or (> 10 mm) [20] Symptomatic stone on mediation [11, 13, 14, 16, 20] Extreme stent-related symptoms [13, 15, 20] Obstructing ureteral stone [11, 12, 14, 20] Obstructed staghorn stone [20] Recurrent UTI on non-obstructing renal stone [13] Stent exchange [11]	Weigh patient’s risk and surgery benefit before treatment Procedures with lower auxiliary retreatments are preferred, e.g., URS over SWL Stentless or stents with strings are encouraged If possible, insert stents and NPT under local anesthesia to spare a ventilator If possible, perform procedures as an outpatient or day surgery To reduce anesthesia time and complications, seniors should do surgery
Low priority or Tier 1–3 [10, 11, 13]	It may cause clinical harm, but it is unlikely	<12 weeks	Stone with well-tolerated stent or NPT [11–14, 16] Bladder stone with recurrent UTI or obstruction [14] Ureteral stent removal [17]	
Postpone or Tier 4 [10, 11, 13]	It is unlikely to cause clinical harm	>12 weeks	Asymptomatic renal stone [11–15] Non-obstructing renal stone [11–15] Non-urgent PCNL procedures [13] Normal renal function [12] No solitary kidney [12] Asymptomatic bladder stone [14] Ureteral stents and NPT exchange [14, 18] Ureteral stent removal [16]

### Non-urgent urolithiasis (intervention after 12 weeks)

Several parameters indicate that surgical intervention should be postponed for more than 12 weeks, because clinical harm is unlikely to occur, including non-obstructing asymptomatic renal stones, normal renal function, PCNL procedures, ureteric stent and NPT replacement, and asymptomatic bladder calculi [11–13, 18]. It is worth noting that most of PCNL indications concern large, obstructive, and infected renal stones. They should not be postponed if there is no lack in unit care capacity. Thus, we should interpret the recommendations of PCNL indications carefully.

## Special situations

### Renal colic

Patients with renal colic should be managed conservatively with appropriate pain control and medical expulsive therapy with close follow-up just in case it becomes an emergency [12, 15]. Recently, concerns about the safety of using nonsteroidal anti-inflammatory drugs (NSAID), e.g., ibuprofen, for treatment of pain have been raised, as it might worse COVID-19 symptoms. No doubt that NSAID is an effective treatment for renal colic. Therefore, we should prescribe NSAID, except for patients with fever or have symptomatic viral infections, acetaminophen should be used instead.

### Stone with stent or NPT

Of note, patients who have renal or ureteric stones with indwelling JJ stent or nephrostomy tube are not at risk of progressive renal function deterioration and stone treatment can be delayed up to 12 weeks [11–14, 16]. However, early surgery may be indicated if patient develops extreme bothersome stent symptoms [13, 15].

### Indwelling ureteric stent (to remove or to postpone)

Stenzel et al. recommended to postpone most procedures for indwelling ureteral stent removal, because for most stents with an indwelling time of 6 to 12 months, removal is simple [16]. Instead, Katz et al. recommended ureteral stent removal as an office-based procedure without delay to avoid stent encrustation, recurrent infections, and annoying stent symptoms that require emergency room visit or hospital admission, as well as to minimize the risk of stents being retained/forgotten [17]. In rare occasions, infections associated with ureteral stents can cause serious illnesses, such as acute pyelonephritis, bacteremia, urosepsis, and even death. Thus, during COVID-19, stentless procedures are encouraged after successful surgery. If inserted, we should consider using stents with strings outside the urethra, to be removed on an outpatient basis [12, 13, 15].

### COVID-19 virus in urine

Urologists usually come into contact with urine during their work. Controversy exists regarding the presence of coronavirus in the urine, and the data are not yet robust. Viral RNA was found only in 6.9% among 66 patients who recovered from COVID-19 infection [27]. On the contrary, Wang and colleagues reported the absence of SARSCOV-2 in 72 tested urine samples [28]. This evidence showed that viral load in urine is not too high and the risk of urine contamination is extremely small, as well as standard sterilization of endourology reusable instruments is considered safe in terms of COVID-19 cross-contamination [12]. Nevertheless, patients with suspicious or confirmed COVID-19 should undergo endoscopy and urethral catheterization carefully, and endo-urologists should be protected completely from infection.

## Urolithiasis and long-standing COVID-19 pandemic

It is now clear that the coronavirus infection is not a temporary major pandemic; nevertheless, it represents a challenging long-standing health healthcare problem. While waiting for a new COVID-19 vaccine or treatment, we should put into consideration that the recommendations made for treatment of urinary stone disease in the early days of the COVID-19 pandemic focused mainly on a short-term crisis situation (12–16 weeks). Thereafter, all urologists will face another major confronts for scheduling the long waiting list of patients and manage more complicated cases in a context of an extended period of pandemic, with a potential of second wave pandemic in many countries.

While the literature evidence of how we restart after lockdown and scheduling the long waiting list of patients is insufficient, we believe that it is difficult to develop recommendations that fit all centers, owing to the major differences between them in terms of patients’ number, healthcare coworkers, urology department facilities, the availability of hospital resources, the total number of beds including ICU capacity, as well as the severity and spread of COVID-19 infection in each country. Thus, urologists should deal with the large cumulative number of cases and schedule OR lists properly to provide maximum patient safety.

## Conclusions

Treatment options for urinary stones have markedly changed during COVID-19 pandemic. Regarding anesthesia methods, the optimal methods are still unknown. As patients with urolithiasis have a wide spectrum of clinical scenarios, judicious treatment decision should be made by endo-urologists according to their surgical priority. Of note, it is recommended to postpone most non-urgent elective procedures, and if there are any alarming indications, emergency intervention is necessary. For intermediate- and low-priority cases, endo-urologists should appropriately weigh the patient’s risk and the benefit of the operation before intervention.

## Data Availability

All articles used in the current review available from the corresponding author on reasonable request.

## Abbreviations

NPT: Nephrostomy tube
RIRS: Retrograde intrarenal surgery
PCNL: Percutaneous nephrolithotomy
URS: Ureteroscopy
USA: United States of America
WHO: World Health Organization
